# Chemoresistance of glioblastoma cancer stem cells - much more complex than expected

**DOI:** 10.1186/1476-4598-10-128

**Published:** 2011-10-11

**Authors:** Dagmar Beier, Joerg B Schulz, Christoph P Beier

**Affiliations:** 1 Department of Neurology, RWTH Aachen, Medical School, Pauwelsstrasse 30, 52074 Aachen, Germany

**Keywords:** glioblastoma, cancer stem cell, temozolomide, chemoresistance, BCNU

## Abstract

Glioblastomas (GBM) are a paradigm for the investigation of cancer stem cells (CSC) in solid malignancies. The susceptibility of GBM CSC to standard chemotherapeutic drugs is controversial as the existing literature presents conflicting experimental data. Here, we summarize the experimental evidence on the resistance of GBM CSC to alkylating chemotherapeutic agents, with a special focus on temozolomide (TMZ). The data suggests that CSC are neither resistant nor susceptible to chemotherapy *per se*. Detoxifying proteins such as O^6^-methylguanine-DNA-methyltransferase (MGMT) confer a strong intrinsic resistance to CSC in all studies. Extrinsic factors may also contribute to the resistance of CSC to TMZ. These may include TMZ concentrations in the brain parenchyma, TMZ dosing schemes, hypoxic microenvironments, niche factors, and the re-acquisition of stem cell properties by non-stem cells. Thus, the interaction of CSC and chemotherapy is more complex than may be expected and it is necessary to consider these factors in order to overcome chemoresistance in the patient.

## Introduction

The role of chemotherapy in the treatment of glioblastoma (GBM) has undergone considerable changes in the last two decades. While alkylating substances such as nimustine (ACNU), carmustine (BCNU), and lomustine (CCNU) have been used since the late 1970s [1,2], the introduction of temozolomide (TMZ) as standard treatment paved the way for a broader use of chemotherapy in the treatment of GBM [3,4]. TMZ, in addition to radiotherapy and surgical resection, improved both the overall survival and the progression-free survival in patients with newly diagnosed GBM [3]. Additionally, its low toxicity has led to TMZ being the first chemotherapeutic agent to be suitable for long-term application over several years, although debate continues on this issue [5,6]. Despite these efforts, the prognosis of patients suffering from GBM remains poor, with a median survival of only 14.6 months [3] and with few patients surviving longer than 5 years [7].

Cancer stem cells (CSC) are postulated mediators of chemoresistance. The CSC hypothesis proposes that tumors are driven by subpopulations of tumor cells with stem cell-like properties, referred to as CSC [8]. It further postulates that CSC later differentiate into rapidly proliferating progenitor-like and more differentiated tumor cells that define the histological features of the tumor entity [8]. An important prediction of the CSC hypothesis is that CSC are more resistant towards radio- and chemotherapy than are rapidly proliferating progenitor cells and differentiated tumor cells. CSC survive intensive oncological therapies and then give rise to tumor recurrences [8].

GBM are a paradigm for the investigation of CSC in solid malignancies. The resistance of GBM CSC towards radiotherapy and chemotherapy has been extensively studied in the last 5 years. Here, we summarize the current knowledge on the resistance of GBM CSC to chemotherapy, with a special focus on TMZ as the current standard of care.

### Introduction - cancer stem cells

For several types of brain tumors, including a subgroup of primary GBM, CSC were found to express CD133. CD133^+^, but not CD133^-^, tumor cells were able to reconstitute the initial tumor *in vivo *when injected into immune-deficient nude mice [9,10]. However, recent reports indicate that this initially proposed model may represent an oversimplification and stem cell-specificity of the epitope detected by the antibody AC133 (i.e. glycosylated prominin, CD133 [11]) has been questioned [12]. GBM cells may acquire CD133 after xenotransplanation [13], conversely CD133^+ ^and CD133^- ^cells within CSC lines may have similar tumorigenic potential [14,15]. In addition, CD133 does not appear to be essential for stem cell-like properties, as subgroups of GBM driven by CD133^- ^CSC have recently been identified [16-18]. Thus, stem cell-specific markers other than CD133 were sought for. In recent years new markers (e.g. CD15/SSEA-1, integrin α6) have been described, but there is no consensus on the optimal markers for CSC in GBM [18-21]. The CSC hypothesis states that tumor relapses are driven by CSC having escaped multimodal therapy. Possible explanations for treatment failure include insufficient drug delivery or the fact that the treatment targets only more differentiated tumor cells (the tumor bulk), while sparing the small subpopulation of CSC (e.g. via CSC specific mechanisms to escape chemotherapy-induced cell death) [8,22,23]. The CSC hypothesis further predicts that only therapies that efficiently eliminate the CSC fraction of a tumor are able to induce long-term responses and thereby halt tumor progression.

However, stem cell-specific therapies, although preventing further growth of the tumor, will not result in an impressive shrinkage of the lesion *in vivo *but in a persisting period of stable disease that may be followed by a late reduction of tumor volume [8]. Because CSC constitute only a rare subpopulation within a tumor, a therapeutic agent selectively depleting CSC will not substantially reduce the overall viability of tumor cells, but may efficiently inhibit their proliferation. In a clinical context, the CSC hypothesis challenges the classical oncological response criteria and questions the evaluation of therapeutic approaches by their effects on the tumor bulk [24].

*In vitro*, these implications raise questions about viability assays used to determine the survival of all tumor cells (e.g. metabolic assays like 3-(4,5-dimethylthiazole-2-yl)-2,5-diphenyltetrazoliumbromide (MTT) or water soluble tetrazolium (WST), apoptosis/necrosis assays like terminal deoxynucleotidyl transferase dUTP nick end labeling (TUNEL) or propidium iodide). Such assays may fail to detect the stem cell-specific effects of a drug. Sequential *in vivo *transplantation experiments are the gold-standard to detect the small population of CSC within the whole of tumor cells [25]. Still, this experimental approach is only feasible to answer specific experimental questions but does not allow screening experiments. Several additional techniques that may help to better investigate the response of CSC to chemotherapy are currently evaluated (e.g. immediate testing of sorted tumor cells, treatment of tumor bearing mice, monitoring of DNA in CSC, mixed culture of CSC and cells without stem cell properties). At the moment, the combination of experiments investigating clonogenicity, stem cell marker expression, and differentiation of tumor cells may constitute a feasible set of screening experiments for stem cell toxicity *in vitro *(summarized in [12]). Recently described new cell culture methods that might help to avoid the above mentioned time-consuming experimental approaches remain controversial and require further scientific evaluation [26,27].

### Introduction - alkylating agents and DNA repair

To date, two groups of alkylating agents are commonly used in the clinic: TMZ (8-Carbamoyl-3-methylimidazo (5, 1-*d*)-1, 2, 3, 5-tetrazin-4(3*H*)-one) and nitrosoureas (BCNU - carmustine, ACNU - nimustine, CCNU - lomustine; referred to as CNUs). In contrast to CNUs, TMZ undergoes rapid non-enzymatic conversion at physiologic pH to its reactive compound 5-3-(methyl)-1-(triazen-1-yl) imidazole-4-carboxamide (MTIC). MTIC is thought to cause DNA damage mainly by methylating the O^6^-position of guanine (primary lesion) which then mismatches with thymine in double-stranded DNA (O^6^G-T) in the first cell cycle after treatment. This mismatch induces futile cycles of mismatch repair triggered by recurrent GT-mismatches resulting in either double strand breaks or a critical recombinogenic secondary lesion. This secondary lesion is assumed to be an apurinic/athymidinic site formed during faulty mismatch repair that blocks replication resulting in either DNA double strand breaks (tertiary lesion), sister chromatid exchange, or other aberrations [28-32]. Thus, not the primary lesions caused by TMZ, but the tertiary lesions formed during faulty mismatch repair, induce cell death of the affected tumor cells. Other mechanisms of action have also been described: TMZ can induce prolonged, p53 and p21^WAF1/Cip1 ^- mediated G_2_-M arrest beginning 2 days after treatment with a small number of cells undergoing apoptosis but most undergoing senescence over a 10-day period. In contrast, TMZ induces temporary G2-M arrest accompanied by only minor changes in p53 or p21^WAF1/Cip1 ^expression in p53 deficient cells [33].

The most frequent sites of DNA base alkylation by CNUs are the N^7^-position of guanine and the N^3^-position of adenine [34,35]. In addition, CNUs mediate their cytotoxic effect by chloroethylation at the O^6^-position of guanine which produces an N^1^-deoxyguanosinyl-N^3^-deoxycytidyl crosslink that is poorly repaired and causes DNA strand break during mitosis. The type of cell death caused by CNU-induced DNA damage is dependent on the p53 status of the tumour cells. In p53 wildtype cells CNUs induce apoptotic cell death while they trigger necrotic cell death in p53 deficient cells [36]. Thus, CNUs are also active if a functional DNA repair system is lacking, whereas TMZ requires a well-established DNA repair system to be effective. The mechanisms of action of TMZ and CNUs suggest that differences in the DNA repair system of CSC and tumor cells without stem cell properties are important to understand any differential sensitivities of CSC to alkylating agents, and how these may arise.

In addition, it may help to understand a putative differential susceptibility of CSC to CNUs and TMZ. To present, three studies have investigated the DNA repair systems in GBM CSC. McCord et al. [37] compared the radioresistance of CSC lines and classical serum cultured cell lines. CSC lines showed a substantially impaired DNA repair resulting in a reduced radioresistance of CSC lines. Within a subset of CSC lines, the CD133^+ ^cells were more resistant to irradiation with 2.5 Gy as compared to CD133^- ^non-stem cells. These data indicate that the mechanisms mediating CSC radioresponse differ from those in the traditional model. The report by McCord et al. complements an earlier report by Bao et al. [22] showing CSC resist radiotherapy due to a more efficient DNA system than non-stem cells. After irradiation, CSC activate the DNA damage checkpoint more effectively than tumor cells without stem cell properties due to the activation of Chk1 and Chk2 checkpoint kinases [22]. However, the differential activation of the DNA repair system in CSC has been questioned by a recent study [38] and it remains to be clarified if the more efficient DNA repair system postulated by Bao et al. [22] constitutes a common property of CSC. Given that TMZ requires an efficient DNA repair (namely the mismatch repair system) to exert its cytotoxic actions, a detailed knowledge of the DNA repair system in CSC as compared to non-CSC will be crucial to help our understanding how TMZ affects the survival of CSC. The functional role of the DNA repair system in CSC and non-CSC may be further complicated by recent data suggesting different molecular subtypes within GBM [39]. Because the subgroups also differ with respect to p53 mutations, the intertumoral heterogeneity will further increase the complexity of TMZ resistance and susceptibly.

### CSC and alkylating agents - results from serum-cultured GBM cell lines

Although results derived from serum-cultured glioma cell lines may be biased by multiple new mutations induced during long-term culture in serum-containing medium [40,41], several authors have described stem cell-like cells within these cell lines [42,43]. Therefore, experimental data collected in the "pre-CSC era" may provide some clues to the effects of TMZ on CSC and the mechanisms of action. The induction of tumor cell death has been considered as the most important contribution of a therapeutic drug to the overall treatment efficacy. Roos et al. [44] showed that TMZ-induced receptor-mediated or mitochondrial apoptosis in glioma cells was depending on their p53 status. Irrespective of the p53 status, after long-term incubation with TMZ (for up to 144 h), dose-dependent apoptosis occurred at late time points starting after 72-96 h. However, even after continuous exposure to TMZ doses of up to 500 μM, at least 30% viable cells remained. Similar results were described by Hermisson et al. [45] investigating the effects of TMZ on a panel of different glioma cell lines. Even high concentrations of TMZ (up to 1290 μM) resulted in the survival of up to 40% of the cells, indicating cytostatic rather than cytotoxic actions of TMZ in these assays. Interestingly, colony-formation seemed to be much more sensitive to treatment with TMZ even after short-term incubation for only 24 h. The EC_50 _values for the prevention of clonogenic growth ranged between 7 μM and 511 μM and correlated with the MGMT status of the cell lines. Hirose et al. [33] treated p53 wild-type cells with 100 μM TMZ for 3 h. The population of apoptotic cells did not exceed 10% but clonogenicity was dose-dependently decreased or completely inhibited. The results of these three publications are representative of multiple reports from glioma and other brain tumor cell lines, reporting almost uniformly a much stronger effect of TMZ on clonogenic growth than on the overall survival of glioma cell lines *in vitro *[33,46-48].

CNUs are more toxic to p53 mutant cells as compared to TMZ. As reported by Roos et al. [36], ACNU-induced cross-links or double strand breaks are repaired in p53 wild-type cells but accumulate in p53 mutant cells. In addition, the repair genes xpc and ddb2 were not up-regulated in p53 mutant cells in response to ACNU indicating a DNA repair defect in the cells causing hypersensitivity to CNUs. On a functional level, colony-formation was much more sensitive to treatment with CNUs than cell viability which did not fall below 30%. In addition, cell death was a late occurring event [36]. Similar findings were reported by Bobola et al. [47], Ashley et al. [49], and Iwado et al. [48]. Together, the majority of reports suggest that CNUs preferentially eliminate clonogenic cells but hardly affect the overall viability. Still, this effect was less pronounced than with TMZ.

In summary, there is a consensus in the literature that the clonogenic growth monitored by colony-forming assays is much more sensitive to treatment with either TMZ or CNUs *in vitro *as compared to the overall cell death *in vitro*. These findings do not suggest an increased resistance of clonogenic cells (including CSC) towards TMZ or other alkylating agents.

However, none of the papers investigated CSC in detail or used assays specific for CSC. As non-stem cells may also show clonogenic growth without being able to proliferate infinitely, the results have to be interpreted with caution [50]. In addition, clonogenic growth is the sum of all events occurring after treatment (including a.o. cell cycle arrest, cell death) and therefore does not allow definite conclusions on the cause of clonogenic cell inactivation.

### CSC and alkylating agents - results from GBM CSC lines

Several recent studies have focused on the effects of chemotherapy on CSC. The CSC hypothesis led to the development of a new cell culture model for GBM. GBM cell lines derived from freshly resected tumor specimens and cultured in serum-free medium supplemented with EGF and bFGF - conditions optimized for the growth of neural stem cells - mirror the phenotype and genotype of primary tumors more closely than serum-cultured cell lines do [40,41]. Thus, experiments with this new cell culture model may yield more valid results on the efficacy of therapeutic agents. The relevance of conventional cell lines (cultured under mainly serum-based media conditions) as means to investigate CSC is disputed. Although the results may be biased by multiple new mutations induced during long-term culture in serum-containing media, these studies are also discussed here. Table 1 gives an overview of studies discussed and indicates the possible technical limitations and key findings of each study.

**Table 1:** Summary of studies investigating CSC and TMZ

Author	serum free medium used?	Key findings (on TMZ and CSC)	Problems
Eramo et al. (2006)	Yes	High resistance of neurosphere cultures to different drugs	No detailed investigation of stem cell properties.

Liu et al. (2006)	Initial culture with serum	Differential susceptibility of CD133^+ ^as compared to CD133^- ^cells	No confirmation *in vivo*, non-physiological TMZ concentrations

Clement et al. (2007)	Yes	Reduced clonogenicity by TMZ.	Study did not investigate TMZ effects in detail.

Ghods et al. (2007)	No	Gliosarcoma cells grown as neurospheres were more resistant to TMZ than the same cells grown as monolayer.	Study did not control for different growth conditions (spheres vs. monolayer).

Chua et al. (2008)	No	Increase of SP cell population after TMZ treatment.	No *in vivo *study on tumorigenicity, serum cultured cell lines (U87).

Beier et al. (2008)	Yes	Depletion of clonogenic and tumorigenic cells by TMZ.	Only a few concentrations investigated.

Bleau et al. (2009)	No (serum-free medium only for neurosphere experiments)	Increased tumorigenicity of glioma cell derived from murine glioma model after long term TMZ treatment (14d). First study that proved that TMZ may increase the tumorigenicity of gliomas.	Murine cells, no information on MGMT methylation status.

Blough et al. (2010)	Yes	Susceptiblity of CSC lines dependent on MGMT expression and promoter status.	No detailed assessement of stem cell properties.

Larmoral-Theys et al. (2010)	No	Decreased tumorigenicity of an oligodendroglial cell line after long-term TMZ treatment.	Serum cultured cell lines; no detailed assessment of stem cell properties.

Glas et al. (2010)	Yes	Differential susceptibility of peripheral and central CSC lines to TMZ. No constant difference between central and peripheral samples.	No systematic assessment of stem cell properties and MGMT status. Conflicting data to Pistolatto et al.

Pistolatto et al. (2010)	Yes	Higher resistance of central, hypoxic CD133 CSC as compared to cells derived from the periphery due to increased MGMT expression.	No validation in a larger set of samples. Conflicting data to Glas et al.

Mihaliak et al. (2010)	Yes	Initial reduction of neurosphere-like growth after TMZ challenge; recovery in 4/5 CSC lines.	No assessment of the MGMT status; only a few concentrations investigated.

Gilbert et al. (2010)	Yes	Initial reduction of neurosphere-like growth after TMZ challenge; inhibition of neurosphere recovery using by NOTCH inhibition.	See comment on Milhaliak et al.

Villalva et al. (2011)	Yes	Decreased clonogenicity after treatment with TMZ.	Study did not investigate TMZ effects in detail.

Hsieh et al. (2010)	Yes	Activation of IGF or shh increases the resistance of CSC to TMZ. Differentiated CSC (with 10% serum) were more susceptible to acute TMZ toxicity than CSC lines.	No controls for different growth conditions (spheres vs. monolayer and serum vs. stem cell medium) mentioned.

Eramo et al. were the first to investigate the chemoresistance of GBM CSC. A marked resistance was observed of GBM CSC lines to different chemotherapeutic agents, amongst them TMZ. GBM CSC lines were treated with 250 μM TMZ for 48 h and minor cell death was determined as a measure of treatment efficacy [51]. Using the same culture model, Clement et al. observed a dose-dependent decrease of proliferation when CD133^+ ^CSC lines were treated with up to 100 μM TMZ, whereas only a minor increase of cell death was observed at similar concentrations [52]. The authors compared the effects of TMZ with the inhibition of the hedgehoc-GLI1 (shh) signaling pathway and found synergistic activity of shh signaling and TMZ. Notably, administration of each of these substances inhibited cell proliferation, although to a different degree. The effects of TMZ treatment on the tumorigenicity of GBM CSC were not investigated. Beier et al. described that TMZ may selectively deplete clonogenic and tumorigenic cells in a dose-dependent manner whereas it hardly affected overall viability. When CD133^+ ^CSC lines derived from primary astrocytic GBM were treated with up to 500 μM TMZ over different periods of time (2 d and 42 d), there was a dose-dependent reduction of CD133^+ ^cells (by 80%), cell proliferation (by up to 100%), as well as clonogenicity and tumorigenicity (by up to 100%), whereas cell death did not exceed 6%. Cells with stem cell-like properties were selectively depleted irrespective of the CD133 or MGMT status. However, MGMT expressing CSC lines required up to 100 fold higher TMZ concentrations. In summary, the authors interpreted their results as selective depletion of CSC from CSC lines by TMZ [53]. Additional confirmatory experiments (e.g. treatment of tumor bearing mice or characterization of tumor material direct after resection) have not been performed. In addition, the authors only investigated three different TMZ concentrations.

In contrast to these reports, Liu et al. used CSC lines that have been established using serum and switched to serum-free culture conditions before the experiments. The authors found that sorted CD133^+ ^CSC showed significant resistance to chemotherapeutic agents (including, amongst others, TMZ). The viability of CD133^+ ^cells was significantly less decreased than the viability of CD133^- ^tumor cells when treated with up to 2000 μM TMZ for 48 h. Notably, even this concentration did not induce cell death in more than 50% of the cells [54]. Ghods et al. investigated chemoresistant and aggressive CSC-like cells within the serum cultured 9L gliosarcoma rat cell line. Viability of 9L cells grown in serum-containing medium as a monolayer and 9L cells grown after serum-deprivation as spheres were compared. The 9L cells grown as spheres were more chemoresistant than cells grown as monolayers. Thus, the authors concluded that CSC were more chemoresistant [55]. Bleau et al. investigated the side population phenotype cells within murine glioma CSC lines derived from PDGF-induced gliomas after TMZ treatment. In line with a report by Chua et al. [56], the number of side population phenotype cells increased, especially in cells lacking PTEN expression, when CSC lines were treated with up to 100 μM TMZ for up to 2 weeks [57]. This translated into an increased tumorigenicity of the long-term TMZ treated cell lines. Unfortunately, neither the MGMT status of the CSC nor the effects of TMZ on stem cell properties were determined. However, the different tumorigenicity of treated and untreated CSC indicated that TMZ increased the number of CSC. Blough et al. investigated the sensitivity of 20 GBM-derived CSC lines cultured under standard conditions [58]. They found that 9 out of 20 CSC lines were susceptible to TMZ, while 11 were resistant. The expression of MGMT, but not methylation of the MGMT promoter, indicated TMZ response *in vitro*. A correlation of CD133 expression and resistance towards TMZ was not reported [59]. TMZ substantially eliminated the proportion of CD133^+ ^stem cell-like cells in a study by Lamoral-Theys et al. using oligodendroglioma HS683 cells. HS683 cells were cultured using serum, but not using medium conditions optimized for CSC. The authors found that long-term treatment of HS683 cells with increasing concentrations of TMZ (from 0.1 μM to 1000 μM) resulted in a substantially decreased tumorigenicity of long-term treated cells, despite a wash-out phase of 8 weeks after TMZ treatment. Notably, the long-term treatment reduced the tumorigenicity and CD133 expression much more effectively than short-term treatment, indicating that the dosing schema influences the depletion of CSC *in vitro*. The MGMT expression remained unchanged in both groups [60]. Villalva et al. investigated effects of STAT3 inhibition and TMZ using CSC lines. They found that both, inhibition of STAT3 and TMZ, substantially inhibited the clonogenic growth of CSC lines and both substances showed a strong synergy [61]. Similar results were reported by Hsieh et al. who investigated the effects of IGF-1 and shh signaling in CSC lines [62].

Based on the high toxicity of TMZ to neurosphere-forming cells within CSC lines, Mihaliak et al. [63] developed a neurosphere recovery assay that was used to investigate the resistance of GBM in the patients. Interestingly and in line with data by Blough et al. [59] and Beier al. [53], they found that the clinically relevant concentrations were able to inhibit the growth of neurospheres in a subgroup of CSC lines and reduced their tumorigenicity. In contrast to the data published by Beier et al., TMZ completely depleted clonogenic cells in only 1 out of 5 CSC lines investigated. NOTCH inhibition successfully inhibited the recovery of neurospheres after successful depletion of clonogenic cells *in vitro *[64].

Two recent studies investigated the susceptibility of CSC derived from the core and the periphery of GBM *in vivo*: Pistollato et al. [65] focused on the effects of the intratumoral hypoxic gradient on the distribution, phenotype and TMZ resistance of CSC. They established and compared CSC lines from the core, the intermediate area, and the periphery of GBM. CSC lines from the periphery showed a higher susceptibility to TMZ induced cell death as compared to CSC from the core. Conversely, cells in the core frequently overexpressed CD133 and MGMT. In summary, the authors postulated that the hypoxic gradient drives the distribution of stem cells. A complementary report by Glas et al. [66] confirmed the differential responses of CSC lines derived from the core and the periphery in a subgroup of GBM, however in most of the samples, there was no uniform difference in the response of the corresponding CSC lines towards TMZ, CNUs, or radiation (alone or in combination).

To present, only one paper has thoroughly investigated the effects of CNUs on CSC lines [67]. Based on an increased expression of the stem cell marker CD133 and a neurosphere-like growth pattern of resistant cells, the authors concluded that BCNU increased the proportion of CSC in the cell lines investigated.

Due to differences in experimental approaches, these studies are difficult to compare. Given that serum-cultured cell lines rapidly acquire multiple mutations and substantially differ from the original tumor, they may provide conflicting results as compared to studies using medium conditions favoring the growth of CSC. However, most recent studies used CSC lines continuously cultured under serum-free conditions. In summary, Eramo et al., Bleau et al., and Pistollato et al. reported an increased resistance of CSC while Beier et al., Bleau et al., Mihaliak et al., Gilbert et al., and Clement et al. described experimental data relating to CSC and TMZ which is consistent with an increased susceptibility of CSC towards TMZ. The data presented by Glas et al., do not allow unambiguous conclusions on the susceptibility of CSC towards chemotherapy (overview in Table 1).

The data presented in the above-mentioned papers allows several conclusions and points towards open questions: (I) All papers uniformly reported that MGMT protein expression is associated with a high resistance of CSC to TMZ. However the role of other factors known to modulate chemoresistance has not been investigated in detail. (II) Whenever investigated in detail, neurosphere-forming cells without MGMT expression were susceptible to TMZ. (III) Different TMZ schedules and concentrations may result in conflicting experimental results. (IV) Environmental factors, like hypoxia in the core of GBM, may contribute to the resistance of CSC towards TMZ. (V) Several signaling pathways, e.g. Shh, IGF-1/PI-3 kinase, NOTCH, or STAT3 interfere with TMZ resistance. (VI) There is no consensus on adequate methodologies to investigate CSC *in vitro*.

### Intrinsic chemoresistance

**Figure 1:**
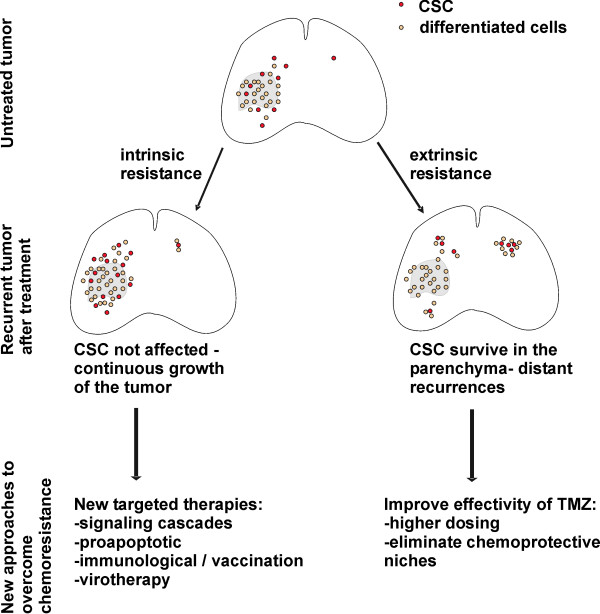
**In GBM with intrinsic resistance, both CSC in the tumor bulk and invading CSC give rise to tumor recurrences after treatment**. In GBM with extrinsic resistance, CSC in the tumor bulk were depleted and recurrences were formed by invading CSC that were protected in the brain parenchyma.

A set of papers (listed in Table 1) on CSC and TMZ focused on the factors modulating chemoresistance of CSC to alkylating agents. *In vitro*, approximately half of all GBM cell lines resisted to TMZ concentrations of 50 μM [45,53,59]. They obviously activated mechanisms of intrinsic chemoresistance (Figure 1). To date, MGMT is the best characterized and the most important modulator of chemoresistance in GBM [68-71]. There is consensus in the literature that MGMT expression substantially increases the resistance of CSC [53,54,59,65]. However, there is conflicting data regarding overexpression of MGMT in the stem cell compartment [53,54]. The expression of MGMT in GBM CSC results in a 10-fold increase of TMZ-resistance [45,53]. The resistance of MGMT expressing CSC [59] fits well to the clinical observations that patients without methylation of the MGMT promoter rarely survive longer than 2 years [69]. In these tumors, all cells may have an obvious intrinsic resistance to TMZ. However, relapsing GBM maintained by CSC without intrinsic resistance to TMZ are difficult to understand, assuming an intrinsic susceptibility of CSC.

The role of multi-drug resistance proteins is controversial. Although expressed in GBM cells, it remains unknown whether TMZ is actually transported by these proteins. Although TMZ incubation increased the proportion of ABCG1 expressing cells (i.e. side population cells) [56], TMZ was not a substrate for the ABCG1 transporter in murine glioma cells [72]. Schaich et al. reported that MDR1 (ABCB1) mediated TMZ resistance and was an independent predictor for TMZ responsiveness [73].

TMZ requires an intact mismatch repair system to cause toxic double stranded breaks. Thus, mutations in the major crucial component of the mismatch repair system mutS homolog 6 (MSH6) mediated TMZ resistance especially in recurrent GBM after TMZ-based radiochemotherapy [74,75]. A well-characterized mechanism of resistance inhibits cell death induced by double stranded breaks. Signalling cascades involved include mutations of p53 and Poly(ADP-ribose)polymerase (PARP) signalling and mutations modulating the apoptotic cascade that executes double stranded break-induced apoptosis [44,76]. Additional but less well-characterized proteins contributing to chemoresistance include protein glutathione-S-transferase. To date, none of these mechanisms of chemoresistance in CSC lines have been investigated in detail.

In addition, the fate of CSC after TMZ treatment *in vitro *and *in vivo *remains unknown, although a loss of stem cell properties in GBM CSC lines and a pronounced sensitivity of colony-formation to treatment with TMZ has been observed in serum cultured cell lines [33,45-48]. In susceptible GBM, TMZ can induce only long lasting cell cycle arrest (G2/M arrest) [33,77], senescence [78,79], or autophagy [80,81]. However, despite the mismatch of minor cell death and massively reduced colony-formation, apoptotic cell death [33,44,48,82] always occurs.

### Extrinsic mechanisms of chemoresistance

Under experimental conditions, TMZ may completely eliminate CSC, suggesting that a selective depletion *in vitro *is feasible [53]. In contrast, the survival of patients treated with TMZ did not stabilize [7] suggesting that residual CSC survived *in vivo *(Figure 1).

The blood-brain-barrier constitutes a specific hurdle for drugs in the brain that reduces the activity of chemotherapy due to decreased concentrations in the brain parenchyma. In approximately 50% of all glioma cells lines, clonogenic cells and/or GBM CSC were highly susceptible to TMZ concentrations of 50 μM *in vitro*. Still, TMZ concentrations of 5 μM did not deplete clonogenic cells *in vitro *irrespective of cell culture model or MGMT status [45,53,59]. The actual concentrations achieved in the plasma and the brain parenchyma of the patients are therefore important to understand if and where within a GBM TMZ concentrations suffice to eliminate tumor cells, irrespective of stem cell properties. The maximum TMZ concentrations in the plasma of patients are well established and range between 27 μM and 50 μM [83]. In contrast, the maximum TMZ concentration in the brain is still unknown. PET studies suggested a concentration of 10-18 μM in the normal parenchyma, however, this methodology does not differentiate between intravasal and interstitial TMZ [84]. In CSF, maximal TMZ concentrations ranged from 0.5-10 μM with most patients achieving concentrations around 5 μM (i.e. 1 μg/ml) [85]. These values were confirmed by a study reporting on interstitial TMZ concentrations of 3 μM [86]. Although detailed studies are lacking, it appears plausible that tumour cells within the contrast-enhancing lesions (i.e. regions of impaired blood-brain barrier) are exposed to concentrations approximating plasma concentrations. In contrast, invading tumours cells are likely to be exposed to concentrations measured in the healthy brain parenchyma (approximately 3-5 μM). Unfortunately, similar studies for CNUs are lacking. Knowing the susceptibility of CSC *in vitro*, these studies strongly suggest that the incomplete penetration of the blood-brain barrier by TMZ may constitute a major factor for chemoresistance of invading CSC. In line with this idea, a recent report analyzing the recurrence patterns of GBM could show that MGMT non-methylated GBM tend to relapse at the site of primary occurrence [87] whereas MGMT-methylated GBM showed a significantly higher proportion of distant metastases. However, this report remains controversial [88] and the concept described does not explain why susceptible GBM also develop recurrences develop from contrast enhancing lesions under ongoing TMZ therapy.

Extrinsic factors of the microenvironment contribute to the chemoresistance of haematological and solid tumors (reviewed in [89]). In these tumors, three different mechanisms of environmentally-mediated chemoresistance have been described: direct cell-cell interactions, local secretion of cytokines like IL-6 or SFD-1, or micro-environmental factors such as hypoxia [89,90]. Although these mechanisms may substantially contribute to chemoresistance of GBM there is only indirect evidence to suggest that these mechanisms may be relevant for GBM CSC. Villalva et al. showed that the activation of STAT3 increases the resistance of CSC to TMZ [61]. Given the expression of IL-6 protein family members like LIF in GBM [91], it is likely that they contribute to chemoresistance of GBM CSC.

### Loss of cellular hierarchy as new mechanism for chemoresistance to TMZ

The CSC hypothesis predicted that the selective elimination of CSC would halt the perpetual growth and tumorigenicity of tumor cells. However, this hierarchy was recently called into question [14,50]. It was suggested that the "stemness" of tumor cells may be induced by niche factors such as hypoxia [65,92-94]. Conversely, these data imply that tumor cells may acquire CSC properties. Several lines of evidence support this concept: Wang et al. were the first to show that CD133-negative tumors may acquire CD133 in a xenotransplantation model [13]. More recently, Chen et al. reported that different types of CSC within one cell line. Within a given cell line, CSC may give rise to other subtypes suggesting a less stringent cellular hierarchy [14]. In melanoma, Morrison et al. showed that these tumors may entirely lack a cellular hierarchy [95]. The generation of new CSC from differentiated cells may therefore represent a new mechanism for therapeutic resistance after selective elimination of CSC [8]. However, additional experimental data is required to confirm this concept.

### Summary and Outlook

There is no consensus in the literature on the effects of chemotherapeutic drugs on GBM CSC *in vivo *or *in vitro*. Still, the available experimental data clearly indicate that resistance of GBM and derived CSC against TMZ is much more complex than expected. The initial predictions of the CSC hypothesis remain true under specific experimental conditions but multiple additional aspects have to be considered to understand why GBM almost invariably relapse. This review summarizes several known aspects of chemoresistance; additional contributors are likely to be discovered. A more comprehensive view will help to better understand the multifaceted factors that contribute to the survival or reoccurrence of CSC causing fatal relapses in the patient.

## Competing interests

CPB has received an unrestricted travel grant from Schering Plough in 2008. All other authors declare that they have no competing interests.

## Authors' contributions

DB, JBS, and CPB wrote and finally approved the manuscript.
